# Registration accuracy comparing different rendering techniques on local vs external virtual 3D liver model reconstruction for vascular landmark setting by intraoperative ultrasound in augmented reality navigated liver resection

**DOI:** 10.1007/s00423-024-03456-z

**Published:** 2024-09-03

**Authors:** Nonkoh J. Sheriff, Michael Thomas, Alexander C. Bunck, Matthias Peterhans, Rabi Raj Datta, Martin Hellmich, Christiane J. Bruns, Dirk Ludger Stippel, Roger Wahba

**Affiliations:** 1 Department of General, Visceral, Cancer and Transplant Surgery, Faculty of Medicine, University of Cologne, University Hospital Cologne, Cologne, Germany; 2 Department of General, Visceral and Oncological Surgery, Helios Hospital Berlin-Buch, Berlin, Germany; 3 Institute of Diagnostic and Interventional Radiology, Faculty of Medicine, University of Cologne, University Hospital Cologne, Cologne, Germany; 4 CAScination AG, Steigerhubelstrasse 3, 3008 Berne, Switzerland; 5 Institute of Medical Statistics and Computational Biology, Faculty of Medicine, University of Cologne, University Hospital Cologne, Cologne, Germany

**Keywords:** Augmented reality, Intraoperative 3D navigation, Innovation, Liver surgery, Registration accuracy, Segmentation

## Abstract

**Purpose:**

Augmented reality navigation in liver surgery still faces technical challenges like insufficient registration accuracy. This study compared registration accuracy between local and external virtual 3D liver models (vir3DLivers) generated with different rendering techniques and the use of the left vs right main portal vein branch (LPV vs RPV) for landmark setting. The study should further examine how registration accuracy behaves with increasing distance from the ROI.

**Methods:**

Retrospective registration accuracy analysis of an optical intraoperative 3D navigation system, used in 13 liver tumor patients undergoing liver resection/thermal ablation.

**Results:**

109 measurements in 13 patients were performed. Registration accuracy with local and external vir3DLivers was comparable (8.76 ± 0.9 mm vs 7.85 ± 0.9 mm; 95% CI = -0.73 to 2.55 mm; *p* = 0.272). Registrations via the LPV demonstrated significantly higher accuracy than via the RPV (6.2 ± 0.85 mm vs 10.41 ± 0.99 mm, 95% CI = 2.39 to 6.03 mm, *p* < 0.001). There was a statistically significant positive but weak correlation between the accuracy (d_Feature_) and the distance from the ROI (d_ROI_) (r = 0.298; *p* = 0.002).

**Conclusion:**

Despite basing on different rendering techniques both local and external vir3DLivers have comparable registration accuracy, while LPV-based registrations significantly outperform RPV-based ones in accuracy. Higher accuracy can be assumed within distances of up to a few centimeters around the ROI.

## Introduction

The aim of oncologic liver surgery is a complete tumor resection. Therefore, relevant anatomical structures and sufficient future liver remnant need to be preserved. In extended liver resections, this is crucial for treatment success. The combined use of resection and thermal ablation can spare liver parenchyma [1–3].

For optimal planning of the resection a unique ability to transfer the liver anatomy into 3-dimensional imagination and understanding is required by liver surgeons. As liver anatomy and vascularity show high variability [4, 5], (pre-) surgical visualization of individual anatomical structures through ultrasound, CT or MRI are essential for patient-specific treatment planning. In addition, intraoperative ultrasound (IOUS) is used to complement the pre-operative diagnostic assessment [6, 7].

Pre-operative virtual 3D image reconstruction of the liver based on two-dimensional CT or MRI images, can improve the planning of the resection [8]. To this end, two-dimensional CT/MRI images of the liver are used to create three-dimensional virtual models using dedicated image postprocessing software tools. These computational models visualize the individual vascular anatomy as well as the exact tumor location, in a process called segmentation, providing segmental anatomy and volume analysis [8, 9]. Based on that information, surgical planning especially for complex liver resection can be facilitated by a technique known as virtual hepatectomy (VH), by which an estimation of the remaining liver remnant volume after resection along selectable resection planes is provided. VH has been shown to lead to improved patient outcomes [10].

### Creation of intraoperative augmented reality and fusion imaging using virtual 3D liver models with IOUS during navigated liver resection

Intraoperative navigation systems transfer virtual 3D images into the operation theatre. A novel navigation technique emerges with the overlay of IOUS with a preoperatively generated virtual 3D liver model (vir3DLiver) to create augmented reality (AR) by image fusion, all done in a process called registration. In this process, anatomical landmarks (e.g. portal vein branches) are selected in a "region of interest" (ROI) on the vir3DLiver and then acquired by an electromagnetically or optically traced IOUS-probe. A computer program then superimposes structures of the vir3DLiver onto the correlating live IOUS image, displayed to the surgeon on a digital screen in order to provide intraoperative guidance [11–13].

During this registration and the following resection/ablation, the overlay or registration accuracy, respectively, as well as the anatomical correctness of the reconstructed vir3DLiver in terms of the precise visualization of the anatomical structures, especially the portal vein branches, are of utmost importance.

### State of the art

Unlike in hard tissue surgery (orthopedics/neurosurgery), 3D navigation and the created AR are still under development in liver surgery and therefore only used in a few centers. Reasons for this are

- insufficient registration accuracy due to liver deformation caused by breathing motion, surgical mobilization and manipulation resulting in a deviation between the pre-operative and the intraoperative spatial relationship of the liver [14, 15]
- the very elaborate process of generating the pre-operative vir3DLiver for intra-operative navigation, often provided by a third-party company.

For the generation of a vir3DLiver, CT or MRI data are transferred to an external service provider. During this process, only a limited amount of data can be transmitted with high service costs and regulatory issues. The commercial service by MeVis Medical Solutions AG is the most widely used service for pre-operative liver segmentation.

Generating the vir3DLiver locally is an alternative to external commercial data processing in this setting [16]. Here, usually all the CT or MRI data collected during a patient’s liver disease course are available. Access to the entire patient’s history and studies of different imaging modalities may be crucial when interpreting the pre-operative images and judging the nature of individual liver lesions. Moreover, metastases may be difficult to detect or even have macroscopically vanished on the preoperative imaging study following neoadjuvant systemic therapy but may still be intended to be included in the volume of resection or ablation [17, 18]. An inclusion of all relevant liver lesions in the vir3DLiver may thus require access to more than just the pre-operative imaging study. Also, interdisciplinary discussion of imaging findings and procedure planning between the surgeon and the dedicated liver radiologist is advisable and may impact the vir3DLiver editing. In addition, local 3D image reconstruction might be cost-effective.

Accurate vir3DLivers are essential for precise AR-navigated liver resection. The CAS-One navigation system uses a vir3DLiver creating AR to navigate the devices for IOUS, tissue dissection and thermal ablation. For this purpose, the devices are marked with optical marker shields. These can be tracked by an infrared camera and transferred to a display system to create an AR overlay – consisting of the vir3DLiver, the surgical device, and the ultrasound image [13].

Intraparenchymal landmark setting with IOUS could optimize 3D navigation accuracy compared to landmark setting on the liver surface [13, 19]. There are still a number of open questions. It is still not clear a) how accurate the reconstructed vir3DLiver in comparison to the real liver needs to be and b) what influence different techniques of 3D liver segmentation will have on the registration accuracy and the creation of AR overlays.

Insufficient registration accuracy is associated with the risk of being unable to correctly identify vessel segments or tumors, which could harm the success of the resection. Besides the precision of the vir3DLiver, registration accuracy might also depend on the anatomical site of the registration process [20]. Finding the right vessel with the IOUS can cause less or higher deformation of the liver parenchyma depending on the vessel’s location and its distance from the liver surface.

**Fig. 1:**
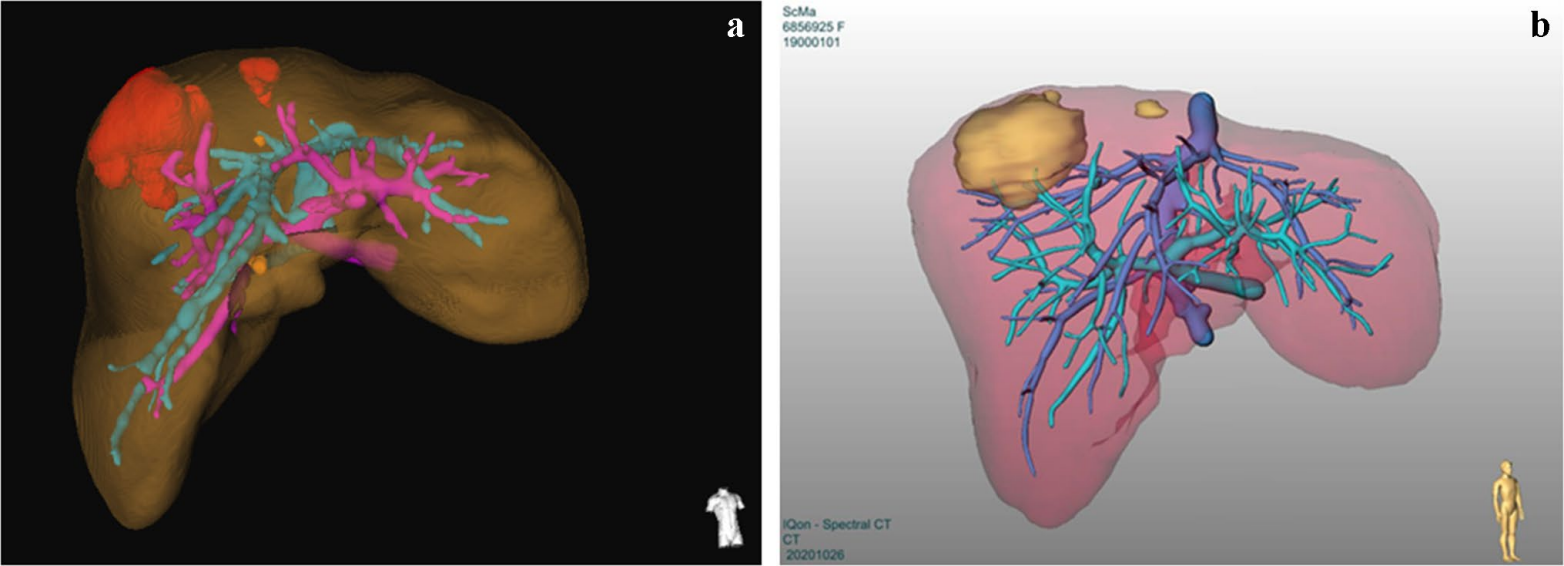
**Virtual 3D Liver Models (Vir3DLivers) a** Vir3DLiver locally generated with Philips’ semiautomatic reconstruction platform IntelliSpace Portal 11. The vessels appear coarser in structure. Brown: liver parenchyma; red/yellow: liver tumor/metastases; magenta: portal vein; turquoise: hepatic vein. **b** Vir3DLiver externally generated by MeVis Distant Services, MeVis Medical Solutions AG. The vessels appear smoother in structure. Red: liver parenchyma; beige: liver tumor/metastases; turquoise: portal vein; blue: hepatic vein

Therefore, this study aimed to evaluate the accuracy of a locally vs externally generated vir3DLiver, and the registration accuracy of an optical infrared-based navigation system when using different anatomical vascular landmarks. In detail, our study aimed at:

- Comparing vir3DLivers generated by local radiologists and surgeons at the University Hospital of Cologne using an application of the IntelliSpace Portal 11 platform (Philips Healthcare) vs commercial, externally provided vir3DLivers by MeVis Distant Services (MeVis Medical Solutions AG). Both programs are semi-automatic liver segmentation tools. However, in both programs the rendering algorithm that generates the vir3DLiver is not known, but it is obvious that during the postprocessing of the digital CT/MRI data, the vir3DLiver reconstruction programs use different rendering techniques creating a,,smoother’’ (MeVis, Fig. 1b) or ,,less smooth’’ (IntelliSpace, Fig. 1a) vir3DLiver surface and vascular structure. How this may affect registration accuracy in AR navigated liver surgery is not clear and thus central question of this work.
- Comparing the use of the left vs the right main portal vein branch (LPV vs RPV) as ROI for landmark setting by IOUS and judging the impact of the distance from the ROI on registration accuracy

## Material and methods

Institutional review board approval for this study was obtained by the Ethics Committee of the Medical Faculty of the University of Cologne (No.: 22–1018-retro).

From April 2019 to March 2021, an optical navigation system (CAS-One, CAScination AG, Switzerland) was used during open liver surgery in 13 patients with HCC or liver metastases who underwent a liver resection and/or thermal ablation at the University Hospital of Cologne, Germany. Retrospectively, the registration accuracy was evaluated via post hoc image analysis.

**Fig. 2:**
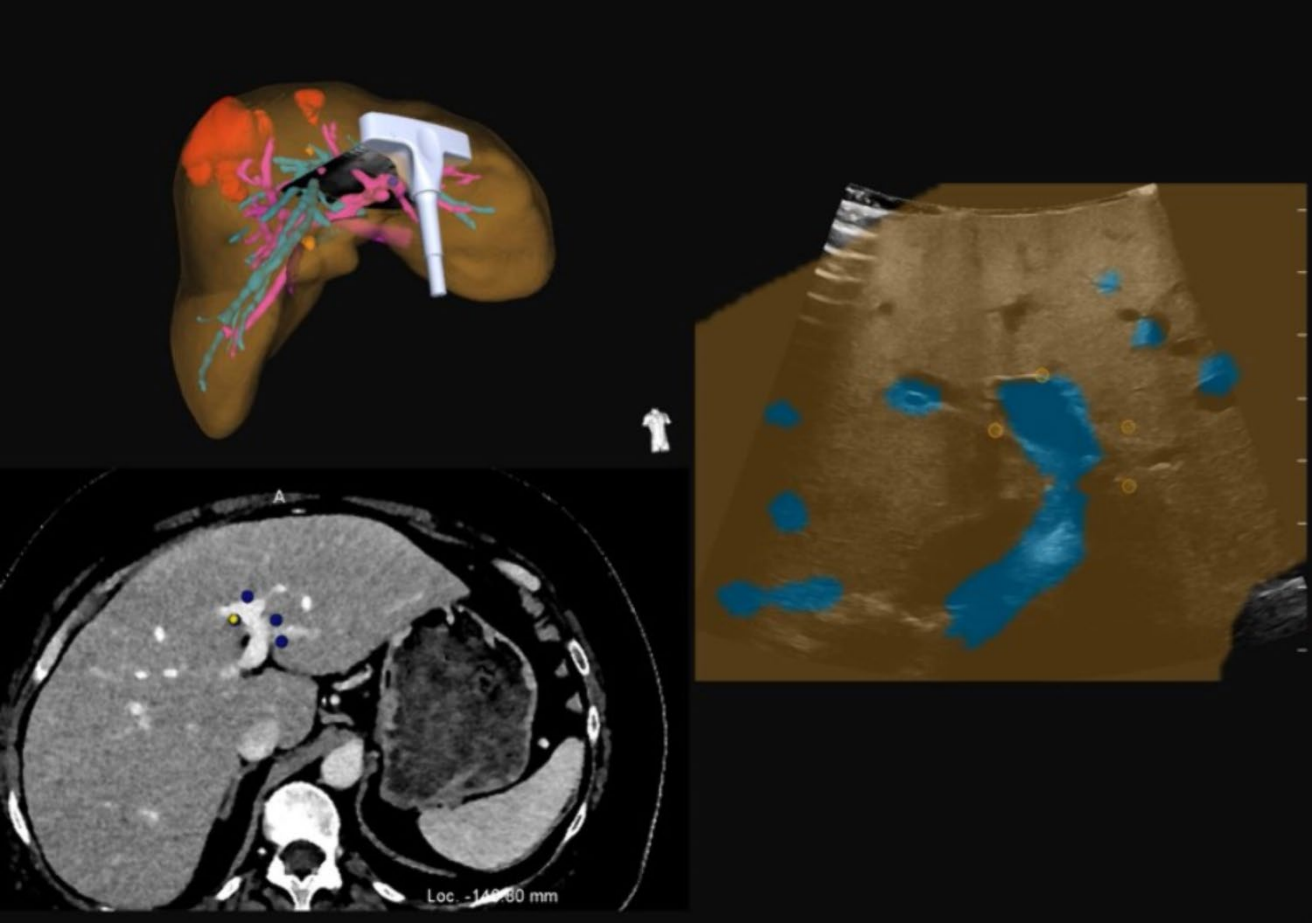
**Screenshot of the intraoperative CAScination Display after the finished registration process via the LPV** Right image: Ubersound Display superimposing the colored vir3DLiver onto the IOUS image in two-dimensional space. Set registration landmarks around the LPV are marked as orange circles. Left upper image: overlay of the IOUS and the vir3DLiver in three-dimensional space. Left lower image: Abdominal CT-Scan with 4 set registration landmarks around the LPV (blue/yellow dots)

CAS-One is a navigation system for open liver surgery and is based on optical tracking technology. It is able to trace marked surgical instruments, such as an IOUS probe or dissection devices, in three-dimensional space using an special infrared camera. Intraoperatively it superimposes the probes as overlays onto the vir3DLiver, the CT- and IOUS images on a touch screen. After registration is finished, the so-called ‘Ubersound’ display computes a two-dimensional overlay of the corresponding vessel sections from the vir3DLiver and the IOUS, creating augmented reality (Fig. 2) [13].

For the intraoperative registration process, two different vir3DLivers were used:

- a locally generated vir3DLiver using a semi-automatic liver segmentation tool, an application of the IntelliSpace Portal 11, Phillips Healthcare (Fig. 1a)
- an externally provided vir3DLiver by MeVis Distant Services, MeVis Medical Solutions AG (Fig. 1b).

For every patient, the registration process was performed separately with the local and the external vir3DLiver. On each model, one registration with the LPV and one registration with the RPV as selected ROI were carried out (four registrations and accuracy measurements per patient).

A registration process was carried out as follows:

First, three to four prominent points around one side of a main portal vein branch (LPV or RPV) were selected on the vir3DLiver and verified on the CT images, displayed on the digital screen. Then, these landmarks were targeted by the optically traced IOUS probe and selected on the IOUS image as well, while the patients were placed under apnea to reduce organ motion. In this way, the internal vascular landmarks were defined using the navigated IOUS probe. These were then used for computing the registration between the virtual liver model and anatomical structures observed in surgery. The registration was then used to display an overlay of the live IOUS image with the vir3DLiver (in the plane of the ultrasound image). The resulting AR display is referred to as Ubersound display. The colored areas seen on the Ubersound image correspond to the 3D model’s vessels. During each surgery, screenshots of the intraoperative landmark acquisition, registration and image fusion process were made for post-operative evaluation and measurement (Fig. 2).

### Measurements

**Fig. 3:**
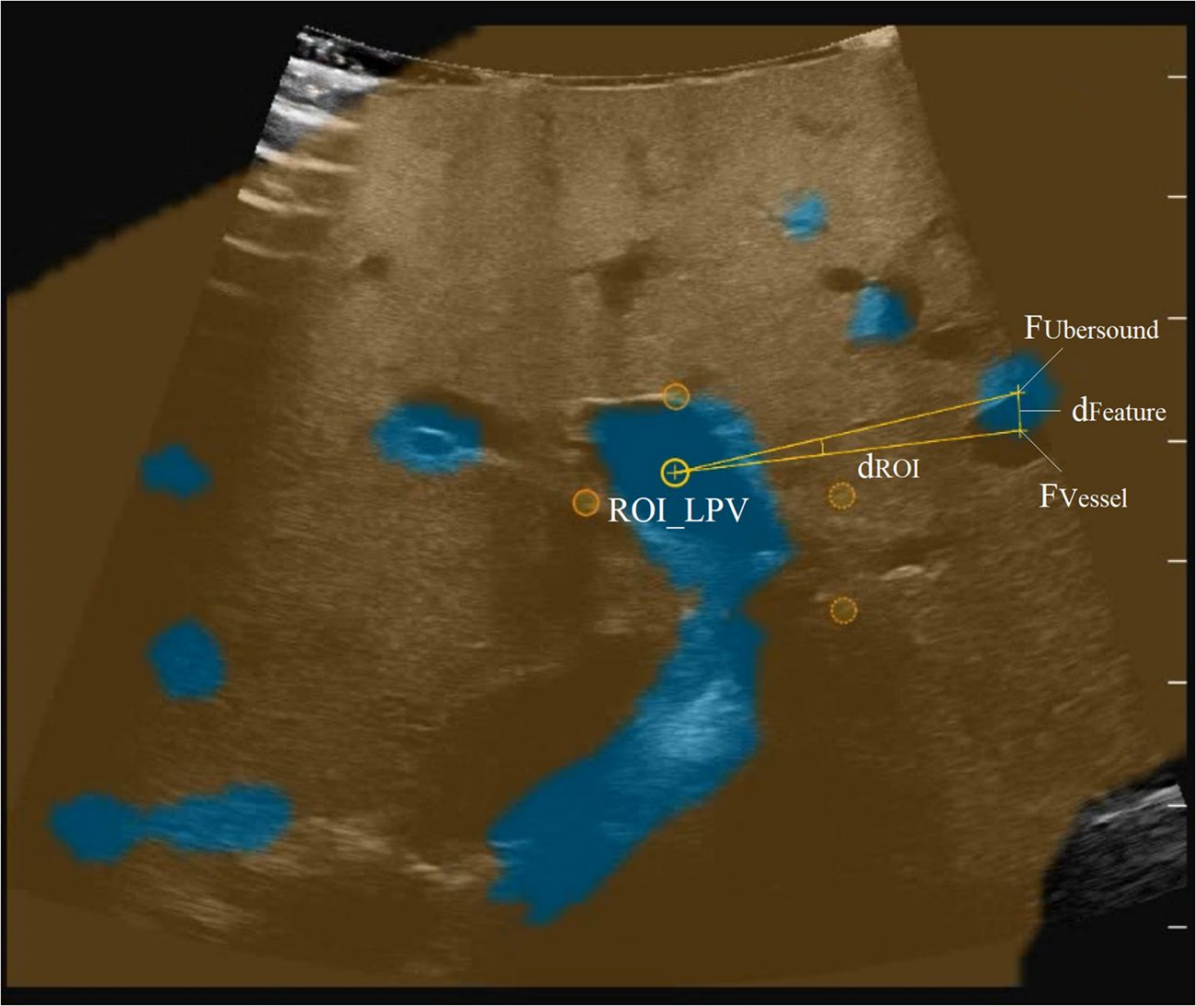
**Measurements done with Fiji (Image J) on a screenshot of the Ubersound Display** Ideally, the 3D model’s blue colored vessel sections (Ubersound) should exactly match with the corresponding vessel sections of the IOUS in plane

The post-operative measurements for this study (Fig. 3) were performed using Fiji based on Image-J (version 1.53c), an evaluated tool for digital image measurement provided by the National Institutes of Health, USA [21, 22]. The object of the measurements were screenshots of the Ubersound display made after the registration was completed.

For each registration, one screenshot was selected for which the exact assignment of the colored 3D vessel sections (Ubersound) to the corresponding vessel sections in the IOUS was possible.

Then the center of the main portal vein branch, chosen as ROI in the registration process before, was defined optically. Here, a point within the overlay of the Ubersound and IOUS vessel section was selected. This point was defined as ROI_LPV_ and ROI_RPV_, respectively. It was chosen in a central position in the IOUS image of the vessel, as positional changes due to deformations are least likely to occur here. Then, outside the ROI, the corresponding vessel sections in the IOUS were identified for as many Ubersounds as possible. d_Feature_ represents the deviation between each one of these. It measures registration accuracy and is the primary outcome parameter in this study. To measure this distance, first two corresponding points (e.g. vessel centers) had to be marked: one in the vessel’s Ubersound, defined as F_Ubersound_, and one in the vessel’s IOUS-image, defined as F_Vessel_. The distance between F_Ubersound_ and F_Vessel_ corresponds to d_Feature_.

To investigate the relationship between the registration accuracy about its distance from the ROI, the distance between the ROI_LPV/RPV_ and F_Vessel_ had to be measured. It was defined as d_ROI_. The measurements for both distances (d_Feature_ and d_ROI_) were taken by using the measuring scale on the right of the Ubersound display (distance between two lines ≙ 1 cm).

In every selected screenshot (four per patient) several measurements were possible, for all corresponding vessels, that could be identified. However, ROI_LPV/RPV_ remained always the same in each screenshot.

### Statistical analysis

Registration data were evaluated by a linear mixed-effects model with random intercept per subject (covariance type VC) and fixed effects for side (LPV vs RPV) and device (local vs external vir3DLiver). Estimated marginal means are stated as mean ± standard error (SE).

To verify whether a possible side difference in registration accuracy between the LPV and RPV can be explained by a different distance of the measured vessels from the ROI, we additionally examined dROI by side.

Differences in marginal means with 95% confidence intervals were calculated to conclude equivalence of the measurement procedures. The correlation of distance from the ROI and registration accuracy was calculated according to Pearson with standard errors determined by bootstrapping, thus taking multiple measurements per patient into account. All statistical calculations were done with SPSS Statistics (IBM Corp., Armonk, NY, USA).

## Results

The intraoperative screenshots of 13 patients were analyzed, resulting in a total of 109 measurements. Of these 109 measurements, 54 were made on registrations based on local virtual 3D liver models** (**vir3DLivers) and 55 based on externally provided vir3DLivers. 70 measurements were performed based on registrations via the left main portal vein branch (LPV) as the region of interest (ROI) and 39 measurements were performed on registrations via the right main portal vein branch (RPV) as ROI (Table 1).

**Table 1:** Distribution of the measurements performed among the different models and sites of the liver

	Local 3D model	External 3D model	Total
LPV	35	35	70
RPV	19	20	39
Total	54	55	109

### Locally vs externally provided vir3DLivers

**Fig. 4:**
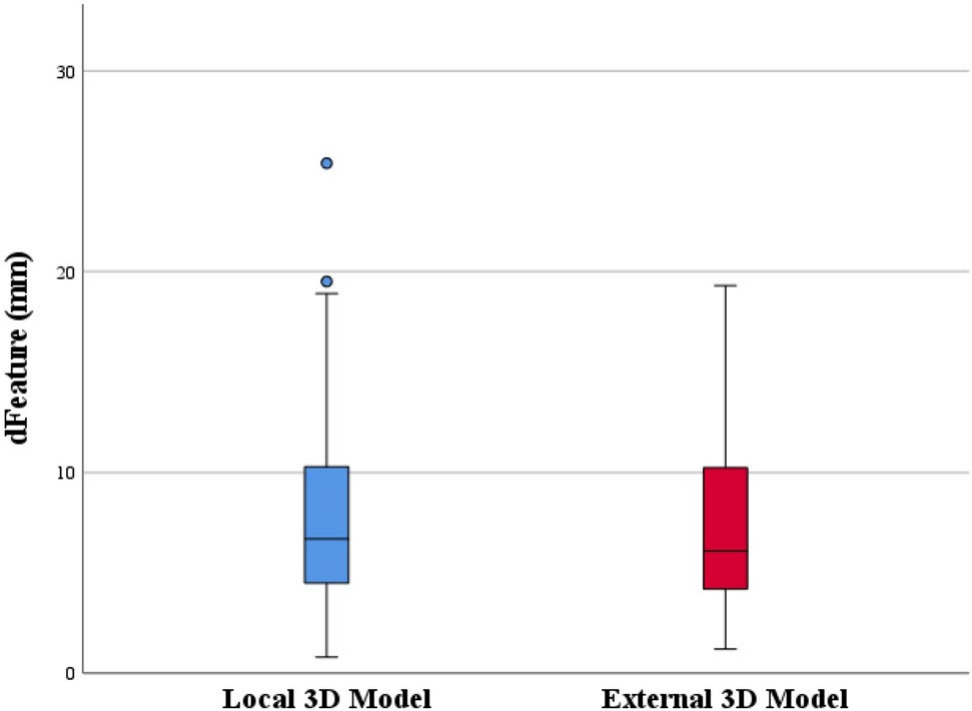
Boxplot 1: Registration accuracy (d_Feature_) in local vs external virtual 3D liver models

The mean registration accuracy was 8.76 ± 0.9 mm (95%-CI = 6.86 to 10.67 mm) with local vir3DLivers and 7.85 ± 0.9 mm (95%-CI = 5.94 to 9.76 mm) with external vir3DLivers. Statistical analysis showed no significant difference in accuracy between both models (mean difference ± standard error = 0.91 ± 0.83 mm; 95%-CI = -0.73 to 2.55 mm; *p* = 0.272) (Fig. 4).

### Left vs right portal vein

**Fig. 5:**
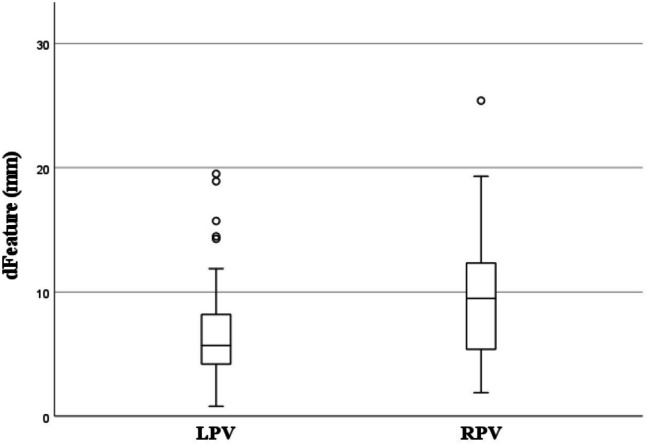
Boxplot 2: Registration accuracy (d_Feature_) via the LPV vs the RPV

The mean accuracy in registrations via the LPV as ROI was 6.2 ± 0.85 mm (95%-CI = 4.36 to 8.05 mm) and 10.41 ± 0.99 mm (95%-CI = 8.35 to 12.47 mm) when using the RPV as ROI, resulting in a mean difference of 4.21 ± 0.92 mm (95%-CI = 2.39 to 6.03 mm; *p* < 0.001). As seen here, there was a significant difference in registration accuracy between registrations via the left and right main portal vein branch in favor of registrations via the LPV as ROI (Fig. 5).

The mean distance of the measured vessels from the ROI (d_ROI_) was 2.08 ± 0.15 cm (95%-KI = 1.74 bis 2.42 cm) and 2.18 ± 0.19 cm (95%-KI = 1.76 bis 2.59 cm) in registrations via the LPV and the RPV, respectively, showing no significant mean difference between both sides (0.09 cm ± 0.22 cm (95%-KI = -0.36 bis 0.55 cm; *p* = 0.68)).

### Correlation between accuracy and distance from the ROI

**Fig. 6:**
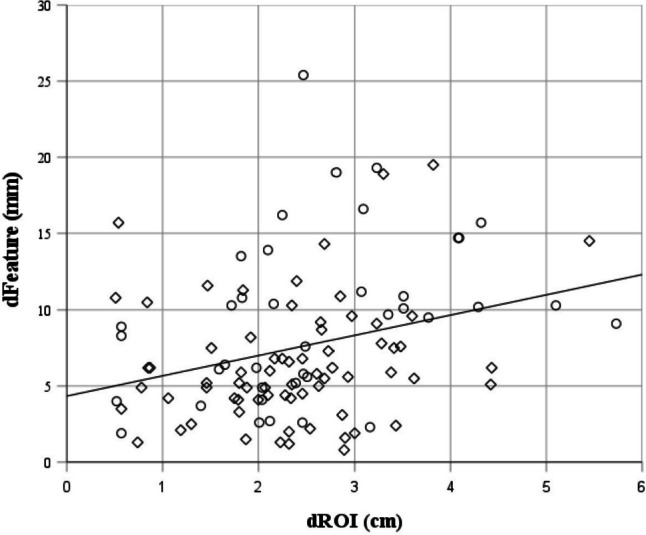
Scatterplot: Correlation between registration accuracy (d_Feature_) and distance from the ROI (d_ROI_) with regression line. ◇: LPV;** ○**: RPV

There was a statistically significant positive correlation between the deviation (d_Feature_, inverse measurement of accuracy) and the fiducial’s distance from the ROI (d_ROI_) (r = 0.298; *p* = 0.002). When visualizing d_Feature_ vs d_ROI_ in a scatter plot, a cluster of better accuracies (< 8 mm) can be observed especially in a range up to 3 cm distance from the ROI (Fig. 6). Quantifying this effect, in a range of 3 cm from the ROI, 70.73% of the measurements resulted in accuracies better than 8 mm.

## Discussion

Augmented reality (AR) aims to improve intraoperative navigation and facilitate liver surgery. Intraoperative fusion imaging, as an overlay between the virtual 3D liver model (vir3DLiver) and intraoperative ultrasound (IOUS), offers a promising AR technology. However, besides the elaborate process of vir3DLiver generation and the ongoing attempt to optimize registration accuracy, it is yet not widely used in the routine. The reconstruction quality of the vir3DLiver, the technique of landmark acquisition, and the anatomical location of the landmarks are important features whenever using AR navigation for the liver.

This study aimed to analyze registration accuracy by comparing local vs external vir3DLivers generated with different rendering algorithms as well as the use of the left main portal vein branch (LPV) vs the right main portal vein branch (RPV) as the region of interest (ROI) for landmark setting by IOUS. Our analysis showed no statistically significant or relevant difference in registration accuracy between locally generated models using the IntelliSpace platform by Philips and external vir3DLivers provided by MeVis. Other than that, there was a relevant discrepancy between registration accuracy depending on the anatomical landmark used for image registration (LPV vs RPV). Registrations via the LPV reached a significantly higher accuracy than via the RPV. About the individual parameters evaluated, there was always found a mean registration accuracy of ≤ 10 mm, which is considered applicable in literature, although smaller values are desirable [13, 23, 24]. Various research groups using fusion imaging in open liver surgery achieved similar accuracies ranging from 4.4 to 11.4 mm [13, 20, 23, 24]. 

This study could demonstrate the local 3D liver reconstruction with IntelliSpace to be equivalent to external reconstruction through MeVis in creating AR via the evaluated 3D navigation system. To our knowledge, it is the first study to examine the effect of the rendering algorithm of vir3DLivers on registration accuracy, showing that different rendering techniques resulting in strongly or less strongly,, smoothened’’ vir3DLivers might not relevantly affect the registration accuracy. Certainly, however, the limits of anatomy must be set for this.

The finding of equivalence of local and external vir3DLivers is supported by Paschold et al., who demonstrated the qualitative comparability of local preoperative 3D segmentation using Synapse 3D (FUJIFILM Europe GmbH, Düsseldorf) and external MeVis reconstructions [16].

One benefit of local/in-house 3D liver reconstruction is that external processing time is avoided, which allows the models to be available faster than with external providers. Besides direct communication between radiologists and surgeons, local reconstruction allows direct access to all CT/MRI images acquired during the course of the patient’s disease. These can provide additional information on tumor development. Especially after chemotherapy metastases may have changed or vanished. These vanished lesions could be targeted with 3D navigation [13, 25]. However, due to the retrospective design of our study, it is unclear if or to what extent additional data have been used in creating the local vir3DLivers.

This study also showed a significantly lower accuracy for registrations via the RPV. Although it is within the scope of clinical applicability (10 mm), even higher accuracy is desired for correct vessel identification and tumor treatment. We could show that this difference was not due to the fiducials being on average farther away from the ROI_RPV_ than from the ROI_LPV_, since the average d_ROI_ was approximately identical on both sides. A reason for the significant side difference between the RPV and LPV might be the greater deformation of the liver tissue on the right than on the left liver lobe while performing the IOUS. The larger distance of the RPV to the liver surface (,,thickness’’ of the right liver lobe), compared to the LPV, might play a central role here. To register the RPV well with the IOUS, sometimes higher compression is needed, which might lead to more deformation. Heizmann et al. investigated in their prospective study the influence of liver mobilization on liver shape and anatomy by comparing preoperative with intraoperative CT-based 3D models after mobilization. They found out that after mobilization alone, the greatest parenchymal deformation (up to 6 cm) occurs in distal regions of the left and right liver (segments 2, 3, and 5,6), while intraparenchymal vascular structures within individual segments deviated relatively little from preoperative imaging (< 0.5 cm). This suggests that registrations should occur locally within the area of the targeted segment. [26] Another factor might be the anatomical variability of the RPV [5, 27, 28], which can make it more difficult to identify prominent landmarks for registration. However, literature is scarce to discuss this issue.

Our findings are confirmed by the results of Satou et al. showing that, due to organ deformation, an acceptable registration accuracy can only be achieved very close to the area of the ROI [11]. Linear regression analysis should provide more detailed information on how accuracy changes with increasing distance from the ROI. Although we found a significant positive correlation (visualized in the scatter plot, Fig. 3), this correlation is not strong (r = 0.298). There might be other factors explaining the scatter of accuracies. Besides the intraoperative deformation of the liver, numerous possible explanations for this exist. Takamoto et al. give a vague indication that the bifurcation level of the measured vessel might also play a role here. [20] However, regarding our data, higher accuracy can be assumed at distances of up to 3 cm from the ROI, which, considering the limited penetration depth of the IOUS of around 10 cm, is a relatively large range, especially as the targeted ROI is often in the center of the image. Ultimately, this result only provides an orientation for interpreting the vascular overlay within a two-dimensional IOUS image in which the ROI is mapped (here the right or left portal vein). The question of how far one can move away from the ROI in three-dimensional space in order to still assume sufficient registration accuracy remains open.

There are some limitations of this study. The measurements were carried out on the Ubersound display and thus represent the positional registration accuracy in a two-dimensional plane. Although the measured vessels of the IOUS and the vir3DLiver correlate with each other, this does not necessarily indicate that these are also in the same segmental planes. The factors depending on ultrasound imaging and interpretation depend highly on the experience of the surgeon. For a more objective determination of registration accuracy, true intra-operative 3D ultrasound would be required. Ribes et al. present a promising method in which, after initial manual registration via anatomical landmarks, registration is performed automatically between in real time segmented 3D IOUS vascular models and preoperative 3D liver models. [29] In the consecutive study by Banz et al., this technique achieves an even higher accuracy compared to manual landmark registration (4.5 vs. 8.4 mm) [13].

Another limitation is the retrospective design of the study. Sometimes it was not possible to reliably assign corresponding vessels based on the screen images for all analyzed images on both virtual 3D models, which explains the different number of measurements. This was more often the case with screen recordings with the RPV as ROI. For all measurements in the Ubersound display, only vessels that were traceably corresponding to each other were used. Despite the lower number of measurements via the RPV compared to the LPV, the statistical results are significant. The 95% confidence interval for the registration accuracy of the RPV includes values ​​of over 10 mm (95%-CI = 8.35 to 12.47 mm), which partly lies in a clinically inapplicable range. If more measurements via the RPV were available, this interval could be further narrowed down, thus providing a more accurate estimate.

## Conclusion

In conclusion, different rendering techniques used in creating the vir3DLivers might not affect the registration accuracy relevantly. The benefit of local image processing and generation of the vir3DLiver is the fast access to the entire patient history, previous imaging studies and the interdisciplinary dialogue between radiologist and surgeon. Using the LPV for landmark setting in the registration process showed a relevant higher registration accuracy than via the RPV.

## Data Availability

No datasets were generated or analysed during the current study.
